# Assessing the mindfulness predictors of mental health: does mindfulness practise or dispositional mindfulness better protect young peoples’ mental health?

**DOI:** 10.1080/21642850.2024.2305723

**Published:** 2024-01-22

**Authors:** Guste Juozelskyte, Jon Catling

**Affiliations:** The School of Psychology, University of Birmingham, Birmingham, UK

**Keywords:** Mindfulness, mental health, wellbeing, depression, anxiety

## Abstract

**Background:**

University is an important time in a young person's life. Although it is a time of exploration, self-discovery and socialising, it is also a time of increased vulnerability to mental health problems such as anxiety and depression. Studies have shown that engagement in mindfulness activities can have a positive effect on mental health. However, research is limited in its scope in particular in relation to stressful (transitional) periods of life.

**Methods:**

The current study aims to address this issue by providing evidence on the predictive impact of both mindfulness practice and ‘dispositional' (or trait) mindfulness on students’ mental health. In total, we gathered data from 190 first-year students.

**Results:**

A multiple regression was utilised for data analysis. This showed that ‘dispositional’ mindfulness, but not mindful activity had a significant impact on mental health scores.

**Conclusion:**

This provides data for future research into the effectiveness of mindfulness interventions for student adaptation to university and potential interventions.

## Introduction

Moving to study at University is a period of increased vulnerability for the development of mental health problems. The transition from a school/college classroom setting to a university environment can be particularly stressful and difficult for first-year students (Briggs et al., 2012). In addition, the COVID-19 pandemic has had a further impact on students mental health in general (Catling, Bayley, et al., 2022; Sahu, 2020). Problems such as stress and depression have been found to reduce attention and ability to focus, negatively impacting students’ academic performance and everyday functioning, and can lead to more serious outcomes such as likelihood of attempted suicide (Liu et al., 2019). Hence, appropriate ways to support student wellbeing should be explored within university programmes, to not only improve student wellbeing and hence academic achievement and university experience, but also to reduce the demand and cost on health and social support services.

Galante and colleagues (2018) assessed whether a mindfulness intervention could improve student resilience to stress. The authors found that after an 8-week intervention (based on a mindfulness course book) students had significantly lower distress scores during an examination period compared to controls who received the usual pastoral support. These findings are further supported by a systematic review of 24 studies using mindfulness interventions which demonstrated that mindfulness programmes could be another effective way of improving students’ mental health (Regehr et al., 2013) and also should be considered for clinical populations (Hofmann et al., 2010). However, these results should be interpreted with caution due to the heterogeneity of the mindfulness interventions, and the low level of representation within the studies, e.g. the vast majority of the sample were female, indicating perhaps that mindfulness practices are still seen as a female activity that is not open to men.

Mindfulness programmes can be both time-consuming and costly which in turn can limit access for university students. In an attempt to address this issue, Flett and colleagues (2020), assessed the benefits of using a mindfulness app, for first-year university students, to reduce distress and ease university adjustment. After a 3 month follow-up, those who used the app more frequently had greater improvements in psychological distress and reported better adjustment compared to non-users. However, the effect was weak due to a high level of inconsistency in the use of the app. It does appear that the evidence on the impact of mindfulness on mental health is mixed. Even if the research finds association between mindfulness (interventions) and better mental health, the question arises as to how these interventions can be adapted to be better accessible for students.

Mindfulness-based interventions (MBIs) are increasingly being delivered via the internet, and there is emerging evidence on the effectiveness of these online MBIs in reducing stress and promoting mental health both in the general population and in a wide range of clinical populations (e.g. Chiesa & Serretti, 2011; Goldberg et al., 2022). In a meta-analysis, Spijkerman et al. (2016) assessed the overall effects of online MBIs on mental health. Fifteen randomised controlled trials were included in their study. A random effects model was used to compute pre–post between-group effect sizes, and the study quality of each of the included trials was rated. Results showed that online MBIs had a small but significant beneficial impact on depression (g = 0.29), anxiety (g = 0.22), well-being (g = 0.23) and mindfulness (g = 0.32), and a significant and moderate effect size for stress (g = 0.51). Specifically, in relation to online MBIs for university students, some previous studies have found that this kind form of training can reduce anxiety and depression (e.g. El Morr et al., 2020). Furthermore, González-García et al. (2021) assessed the feasibility of a brief (16 days) online Mindfulness based intervention to improve mental health among first-year university students during COVID-19 home confinement. Their study showed that after the intervention, stress and anxiety levels had decreased significantly. In further support of these findings, a number of recent review studies have also shown that MBIs can improve mental health specifically in undergraduate students (e.g. Chiodelli et al., 2022; Halladay et al., 2019).

Up until this point the focus has been on the potential impact of mindfulness techniques and practise on mental wellbeing. However, another aspect of mindfulness that can have a positive impact is that of Mindful thinking (or ‘dispositional’ Mindfulness), often referred to as a trait of Mindfulness and associated with an individuals’ frequency of open and receptive attention to and awareness of ongoing events and experiences. Some studies have shown a link between dispositional mindfulness and depression. For example, Yu-Qin Deng et al. (2014) examined the relationship between wandering mind, depression and mindfulness. Their results revealed that the wandering mind was not only positively associated with depression, but also negatively related to dispositional mindfulness. Notably, they also found that Depression was negatively related to dispositional mindfulness. Ayhan and Kavak Budak (2021), in a sample of 151 patients with depression found a statistically negative strong correlation between mindfulness and negative automatic thoughts of patients with depression. However, not all research has shown this association, Van Dam et al. (2011) compared the ability of the Self-Compassion Scale (SCS) and the Mindful Attention Awareness Scale (MAAS) to predict anxiety, depression, worry, and quality of life in a large (*N* = 504) community sample seeking self-help for anxious distress. They found that self-compassion was a robust and significant predictor of symptom severity and quality of life, whereas dispositional Mindfulness was not.

There are only a handful of studies that specifically focus on the relationship between dispositional Mindfulness and depression within a student cohort, and often these have mixed findings. For example, Schut and Boelen (2017) assessed dispositional Mindfulness and depression in a sample of 208 Dutch students at two time points 12 months apart. At timepoint one, they found that dispositional Mindfulness was not associated with depression, whilst at timepoint two it was, suggesting only a tenuous or mutable link between the two. Furthermore, Song (2011) demonstrated that in a small cohort of Korean nursing students that dispositional mindfulness was significantly negatively correlated with depression (*r* = −.73). Finally, Masuda and Tully (2012) investigated whether dispositional mindfulness and psychological flexibility uniquely and separately accounted for variability in depression. Their sample was an ethnically diverse, nonclinical sample of American college undergraduates (*N* = 494, 76% female). They found that psychological flexibility and mindfulness were positively associated with each other, and tested separately, both variables were negatively associated with depression. Furthermore, their results also revealed that psychological flexibility and mindfulness accounted for unique variance within depression.

The current study aims to assess the distinct impact of dispositional Mindfulness and mindfulness practice on depression and general anxiety disorder in a UK-based student sample during their transition to University, and to the authors’ knowledge is the first study to do so.

## Method

### Participants

In total, 190 psychology undergraduate students from the University of Birmingham took part in the study. The final sample consisted of 150 females, 37 males and 3 unspecified gender participants from a non-clinical population. Participants age ranged from 18 to 23 (*M_age_* = 18.7). Participants were recruited through the Research Participation System (RPS) in exchange for research credits.

### Measures

#### Dispositional mindfulness

The Mindful Attention Awareness Scale (MAAS; Brown & Ryan, 2003) is a 15-item single-dimension measure of trait mindfulness items and has high internal consistency, *α* = .92 (Brown & Ryan, 2003). The MAAS measures the frequency of open and receptive attention to and awareness of ongoing events and experiences. Response options ranged from 1 (almost never) to 6 (almost always). Example items include ‘I find it difficult to stay focused on what’s happening in the present,’ ‘I could be experiencing some emotion and not be conscious of it until some time later,’ and ‘I rush through activities without being really attentive to them.’ One item was modified to make it appropriate for adolescents: we changed the item ‘I drive places on “automatic pilot” and then wonder why I went there’ to ‘I go places on “automatic pilot” and then wonder why I went there.’ Item scores were reverse-coded making higher scores indicate a greater degree of mindfulness. To control for social desirability, respondents are instructed to respond to the MAAS in a way that reflects their actual experience rather than in a way they think their experience should be.

## Mindful activities

An additional item was added to assess mindful practise: ‘Do you engage in meditation or mindfulness activities?’ this was assessed on a 6-point Likert scale – Almost always, very frequently, somewhat frequently, somewhat infrequently, very infrequently, Almost never.

## Mental health- depression and anxiety measures

***Depression****:* The Personal Health Questionnaire (PHQ-9) was used to assess self-reported symptoms of depression (Kroenke et al., 2001). The measure includes nine statements asking participants how frequently over the last two weeks participants have been bothered by a number of problems (e.g. ‘Feeling down, depressed or hopeless’) and is measured on a 4-point Likert scale from 0 – ‘Not at all’ to 3 – ‘Nearly every day’. Scores can range from 0 to 27, with higher scores indicating more depression symptoms. The PHQ-9 is well-established within research and health settings and has high sensitivity and specificity when testing in clinical settings (Gilbody et al., 2007). Internal reliability for this measure was *α* = 0.72.

**Anxiety:** The Generalised Anxiety Disorder (GAD-7) questionnaire was used to measure self-reported symptoms of anxiety (Spitzer et al., 2006). The GAD-7 is made up of seven statements asking participants ‘Over the last 2 weeks, how often have you been bothered by the following problems?’ (e.g. ‘Worrying too much about different things’). The GAD-7 can range from 0-21, with higher scores indicating more anxiety. The measure has high validity and reliability in both patient populations (Kroenke et al., 2001) and the general population (Lowe et al., 2008). The GAD-7 demonstrated good internal reliability at *α* = 0.79.

### Procedure

The questionnaire was created using Qualtrics software and was available on the Research Participation Scheme system for students to participate as part of their module requirement. First, participants were introduced to the study, followed by a consent form which was mandatory to complete to proceed. Basic demographic information such as age, gender, year of study was taken before undertaking the questionnaire. Participation was voluntary and those who decided to participate were rewarded RPS credits. In conclusion, participants were debriefed.

### Ethical consideration

It was mandatory for participants to sign the consent form before proceeding with questionnaires. Participants were informed that all data would be kept confidential, and their identity would be kept anonymous.

We acknowledge that some questions on the mental health measure appear sensitive thus it was made clear to participants they were not obligated to answer questions they did not feel comfortable answering. Accessible support services were outlined at the end of the debrief. The right to withdraw was available for 48 h post completing the questionnaire.

Ethical approval has been provided by the University of Birmingham, STEM ethics committee (code: ERN_20-1093).

## Data analysis

Multiple linear regression was undertaken using SPSS v27. There was no missing data. An enter ‘method’ was utilised for all regression analyses. A correlation analysis was undertaken for both of the mindfulness measures to check for collinearity.

### Results

Data from 190 participants was utilised for the analysis. Linear regression analysis was used to assess whether dispositional mindfulness, and mindfulness activity could predict anxiety and depression scores.

## Depression measure

Linear regression was used to assess dispositional mindfulness and mindfulness activity as predictors of depression. The overall model of regression was significant, Adj *R*^2 ^= .186, *F*(2,189) = 22.55, *p* < .001. Results of the regression analysis indicated that mindfulness was a significant predictor of depression, *t* = 6.71, *p* < .001, Those who scored higher on the dispositional mindfulness measure, scored lower on the depression measure. However, the regression analysis showed mindfulness activity was not a significant predictor of depression (*t* = .411, *p* = .681).

## Anxiety measure

Linear regression was used to assess dispositional mindfulness and mindfulness activity as predictors of anxiety. The overall model of regression was significant, Adj *R*^2 ^= .139, *F*(2,189) = 16.20, *p* < .001. Results of the regression analysis indicated that dispositional mindfulness was a significant predictor of anxiety, *t* = 5.64, *p* < .001, Those who scored higher on the dispositional mindfulness measure, scored lower on the depression measure. However, the regression analysis showed mindfulness activity was not a significant predictor of anxiety (*t* = .33, *p* = .739).

## Correlation and collinearity

A correlation analysis between the two mindfulness measures showed no significant correlation between the two (*r* = .078, *p* = .285). Additionally, the regression analysis showed the collinearity tolerance to be .99 and VIF value as 1.006 indicating very low collinearity.

## Discussion

The aim of this study was to assess whether, dispositional mindfulness and mindfulness activity could predict mental health scores. The results of our analysis show that only dispositional mindfulness was a significant predictor of depression and anxiety scores. Mindfulness practise was not a significant predictors of mental health scores. Participants that indicated they were more mindful, scored lower on the generalised anxiety disorder and depression measures.

The finding that dispositional mindfulness was a significant predictor of depression and anxiety in a student cohort expands on the findings of both Song (2011) and Masuda and Tully (2012), but specifically in a UK population and during a period of transition. Whereas, the finding that mindfulness practices did not predict depression or anxiety in our sample was a surprise, and runs contrary to a range of studies that found that mindfulness training could reduce anxiety and depression (e.g. Chiodelli et al., 2022; El Morr et al., 2020; González-García et al., 2021; Halladay et al., 2019).

It should be noted that one of the limitations of our study was the gender bias within our sample. This bias was caused by the selection process, as this was a self-selecting sample from a student population that was approximately 80% female, so the sample was indeed representative of the student cohort, but not of young people as a whole. This does mean that we have to be cautious when applying our findings across the broad aspect of young people, as (and we think it unlikely, as there is no previous evidence to suggest this) our results may be applicable to just young females. A second limitation is the amount of detail that was gathered on the individuals’ Mindful activities. Whilst we did gauge if participants engaged in any form of ‘mindful’ activity, we did not measure the type of activity (e.g. using mobile Apps or in-person activities/therapy) or the duration of their activities. This omission does feed into our suggested directions for further research (see below).

The most important question that comes from our study is, given the previous research within this sphere, why does dispositional Mindfulness but not mindful practice predict depression and anxiety? Importantly, our analysis showed no significant correlation between these two variables or any co-linearity between them within the regression analysis, so this could not account for our results. It may be that in previous cross-sectional comparisons that dispositional Mindfulness was not a variable that was controlled for, and hence could have acted as a confounding variable within the study analysis. This does have ramifications for potential interventions using mindfulness in that it would appear that it is a mindful ‘outlook’ or ‘disposition’ that needs to be targeted. Given that some view this form of mindfulness as a trait (e.g. Yu-Qin Deng et al., 2014), this may be difficult to change and modify.

Furthermore, some models of mindfulness (e.g. Shapiro et al., 2006) explored a range of relevant literature and found that the mechanisms of Mindfulness could be broken down into distinct ‘axions.’

‘On purpose’ signifies intention,

‘Paying attention’ refers to attention,

‘In a particular way’ encompasses attitude (mindfulness qualities).

They go on to suggest that these Axioms, as foundational elements, give rise to other phenomena, and hence an understanding of these components provides insights into how mindfulness operates. Notably, intention, attention, and attitude are not distinct processes or stages; they are interconnected facets of a singular cyclical process occurring simultaneously. Mindfulness manifests as this continuous, moment-to-moment process. It is fair to draw from this model that these axions are closely linked to habits of behaviour, and we know that active habits take time to develop, and only through repetition and effort do they become behaviours that one can perform with little or no thought. This would suggest that, for example, an App based mindfulness session once a week is not likely to change levels of fundamental dispositional mindfulness.

However, according to Kiken et al. (2015), a heightening state of mindfulness in regular meditation practice over time can increase trait (dispositional) mindfulness. They prospectively examined individual trajectories of state mindfulness in meditation during a mindfulness-based intervention in relation to changes in trait mindfulness. Each week during an eight-week intervention, participants reported their state mindfulness after a brief mindfulness meditation. Participants also completed pre and post intervention measures of trait mindfulness and psychological symptoms. Tests of combined latent growth and path models suggested that individuals varied significantly in their rates of change in state mindfulness in meditation during the intervention, and that these individual trajectories predicted pre–post intervention changes in trait mindfulness. These findings support the idea that increasing state mindfulness over repeated meditation sessions may contribute to a more mindful disposition. It would seem from this study that an intensive, face-to-face, relatively extended intervention could have an impact on ‘dispositional’ Mindfulness, and hence could improve mental wellbeing.

Incorporating these forms of mindfulness education to university programmes could help students transition to university and help mental wellbeing. However, the nature of the intervention would probably make it impractical and economically unviable for most institutions. Hence further research should be squarely focused on finding other more practical, simple and accessible interventions, that also have this positive effect on ‘trait’ or ‘dispositional’ Mindfulness. It does seem intuitive that having a more Mindful outlook and perception of life would have a positive impact on student wellbeing, and sadly we find this lacking in the majority of students cohorts today (e.g. Catling, Michail, et al., 2022).

## Conclusion

The current study demonstrates that the mental well-being of young people is impacted by their own unique level of dispositional mindfulness, but not by their mindful activity. In some ways, this is disappointing as it suggests that ‘mindful’ intervention in the main will not be effective at improving well-being for young people; perhaps these forms of intervention may have a short-term impact but may not transfer to a more general change in thinking. However, there is some evidence that certain forms of more intense one-to-one mindful interventions/techniques may indeed increase an individual’s dispositional Mindfulness and hence may well improve overall well-being. We suggest that further research should assess the different forms of mindfulness activities/techniques/interventions, to assess which can have an impact on dispositional mindfulness and hence which (according to our findings) are more likely to impact on overall mental well-being.

## Data Availability

All data will be provided on request.
